# The Influence of Neem Oil and Its Glyceride on the Structure and Characterization of Castor Oil-Based Polyurethane Foam

**DOI:** 10.3390/polym13122020

**Published:** 2021-06-21

**Authors:** Yi-Han Liao, You-Lin Su, Yi-Chun Chen

**Affiliations:** Department of Forestry, National Chung-Hsing University, 145 Xingda Rd., South Dist., Taichung City 402, Taiwan; g108033204@mail.nchu.edu.tw (Y.-H.L.); g107033204@mail.nchu.edu.tw (Y.-L.S.)

**Keywords:** bio-based polyols, castor oil, foams, neem oil, polyurethane, transesterification

## Abstract

Neem (*Azadirachta indica*) oil is a non-edible oil that contains azadirachtin, which can be used as a biopesticide. This study synthesizes bio-based polyurethane (PU) foam from neem and castor (*Ricinus communis* L.) oil at normal temperature and pressure. Neem oil can be reacted to narrow-distribution polyol by transesterification of oil and glycerol. Neem oil glyceride (NOG) can be used as polyol for bio-based PU foams and can be blended with castor oil homogeneously to reduce the cost of production. The composition of polyol was castor oil and 0 to 20% molar ratios of NOG. Hexamethylene diisocyanate trimer (Desmodur N) was used as isocyanate. The molar ratios of NCO/OH were set as 1.0, 1.5 and 2.0. The average hydroxyl contents of castor oil, neem oil and NOG were 2.7 mmol/g, 0.1 mmol/g and 5.1 mmol/g, respectively. The reaction time of bio-based PU foam could be adjusted between 5 to 10 min, which is acceptable for manufacturing. The densities of PU foams were between 49.7 and 116.2 kg/m^3^ and decreased with increasing NCO/OH and NOG ratios and decreasing neem oil. The ranges of specific compressive strength of foams were from 0.0056 to 0.0795 kPa·m^3^/kg. Increasing the NOG and neem oil ratio significantly enhanced the specific compressive strength in the low NCO/OH ratio. The solvent resistance and thermogravimetric (TG) results showed that the foams have high water and thermal stability. NOG can help to increase solvent resistance. Adding neem oil reduces the solvent resistance. The results indicated that increasing NCO/OH and NOG ratios increases the cross-linking density and hard segment content of PU foams. This investigation demonstrated that castor oil-based PU foams are improved by adding NOG to the polyol mixture. PU foam has excellent properties. Neem oil can be used in manufacturing processes to produce high-performance foams via a green synthesis process.

## 1. Introduction

PU resin has flexibility properties and suits to use as various products [1,2]. These raw materials of commercial PU resins are produced from petroleum. However, petroleum is a limited resource with environmental concerns. Chemicals derived from renewable source have attracted attention. The most widely used renewable source to manufacture raw materials for PU products is vegetable oil, available in abundance [3]. Previous investigations have reported bio-based polyols by derivative various vegetable oils, such as neem, castor, rapeseed and soybean oils to develop bio-based PU foams [3,4,5,6]. The plant oils can be divided into food and inedible crops. Edible plant oil is not only high production but also stable supply as a positive aspect. On the other hand, Edible plant oil uses in non-food applications and would result in harm to food supplies and biodiversity. Nowadays, non-edible plant oil has used as a chemical to replace fossil-based resources [7,8,9,10].

Castor oil, extracted from the beans, is a toxic oil, which is no competition with plant oil consumption of food crops. The main components of castor oil is ricinoleic acid (12-hydroxy-9-*cis*-octadecenoic acid, C_18_H_34_O_3_), which accounts for ca. 89% directly as a polyol because of the intrinsic hydroxyl groups [11,12,13]. Moreover, castor oil-derived polyols with high functionalities can help to enhance the compressive strength of the PU foam [14]. The stable supply helps make castor oil a widely used raw material for industrial applications [15]. Neem (*Azadirachta indica*) oil is also a non-edible oil extracted from seeds. Neem, a *Meliaceae* family plant, is distributed in tropical countries such as India, Myanmar and Malaysia. In 1959, neem was the only plant that survived the locust infestation in Sudan. Investigation indicated that neem has azadirachtin, which is an active compound against bacteria, fungi and parasites. Therefore, neem products have long been used for herbal medicine, pesticides and antibacterial agents. Unsaturated fatty acids account for about 55% of the content of neem oil. The fatty acid composition includes oleic acid (50%), stearic acid (30%), palmitic acid (12%), linoleic acid (5%) and arachidic acid (3%). The fatty acids can be modified by acetylation or transesterification and reacted with isocyanates to produce PU resins [16]. Chaudhari et al. (2013) produced renewable source-based PU coatings from polyetheramide prepared from neem oil with cyclohexanone and tetrahydrofuran. The impact resistance, flexibility, adhesive properties and chemical resistance showed excellent performance for PU coatings. The thermal stability of neem oil amide based PU coatings was shown to be better than those of traditional urethane and alkyd coatings [17]. Marathe et al. (2015) modified neem oil into acetylated polyester polyol, which responds with different kinds of isocyanates to prepare PU materials with xylene. The results showed that the corrosion resistance of PU materials improved [18]. Das (2021) prepared neem oil-based PU resins by the alcoholysis–polyesterification process with methanol. The PU resin also has application potential as an anticorrosive coating [19]. Previous studies demonstrated that neem oils have a high potential to replace fossil-based polyols for manufacturing high-performance PU coatings. However, the synthetic route of neem oil-based PU resin use toxic organic solvent. The price of neem oil (ca. 19 USD/L) is much higher than that of castor oil (ca. 7 USD/L) in Taiwan. Furthermore, no studies previous have prepared neem oil as the raw material of PU foam. 

**Figure 1:**
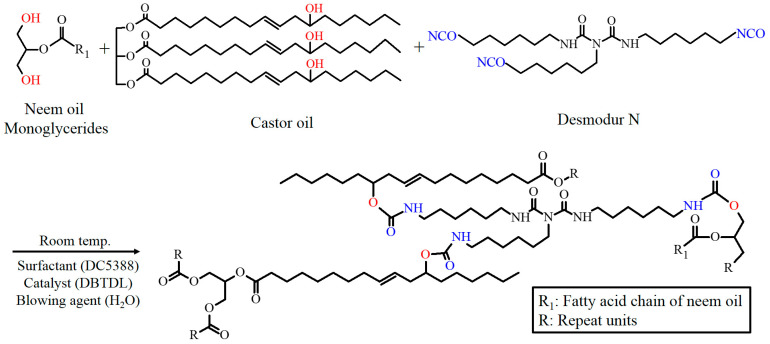
Chemical synthesis route for bio-based PU foam.

This study is the first investigation using neem oil as a bio-based polyol for preparing PU foams, to the authors’ best knowledge. Castor oil was used as the main bio-polyol to reduce the cost. NOG was prepared by transesterification of oil and glycerol to introduce the hydroxyl group. This study used castor oil and NOG as polyols to react with isocyanate (Desmodur N) and produce bio-based PU foams. Neem oil also replaced the NOG as a natural modifier for preparing reference PU foam [20]. The previous studies have demonstrated that diisocyanates had acute and chronic toxicities [21,22]. Therefore, an isocyanate trimer was used in this study. The mixture of raw materials was blended and reacted under room temperature directly. The solvent-free synthetic route shows in Figure 1. The hydroxyl groups of castor oil and NOG reacted with NCO groups of Desmodur N to form a urethane structure. The neem oil-based PU foam was prepared as a reference to compare the results. The optimal condition of PU foams was confirmed from chemical, mechanical and thermal results. 

## 2. Materials and Methods

### 2.1. Materials

Castor and neem oil were obtained from Chung-Hsing Chemical (Taichung, Taiwan) and Darwin Biotech Co., Ltd (New Taipei City, Taiwan), respectively. Isocyanate Desmodur N were obtained An Fong Develop Co., Ltd. (Taichung, Taiwan). Desmodur N has a 23.7% NCO content. The NCO content of isocyanate was determined in the authors’ laboratory [23]. Organo-siloxane (DC5388, DABCO) and dibutyl tin dilaurate (DBTDL) were purchased from Ya Chung Industrial Co., Ltd. Glycerol and calcium oxide (CaO) were purchased from Union Chemical Works LTD and Sigma Aldrich, respectively. 

**Figure 2:**
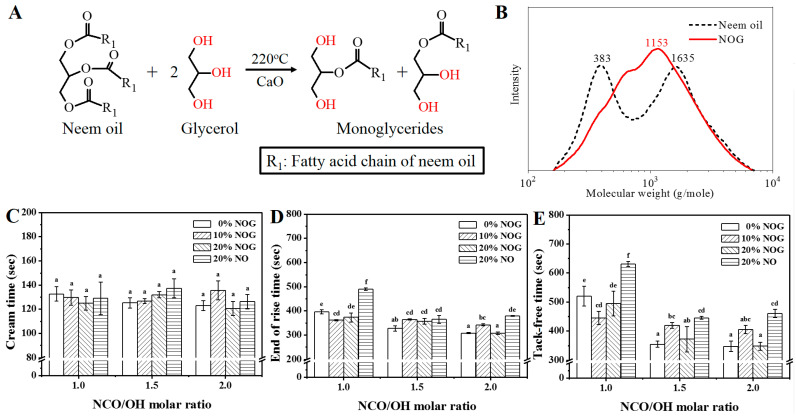
(**A**) General transesterification scheme of neem oil [14]. (**B**) Molecular weight distribution of neem oil and NOG. Cream time (**C**), end of rise time (**D**) and tack-free time (**E**) of PU foams. The NCO/OH molar ratios of O1-2, O2-2 and O3-2 set as 1.3, 1.9 and 2.5, respectively. The data analyzed using one-way analysis of variance (ANOVA) with Scheffe’s test, the different lowercase letters indicate statistical differences in the bar graph.

The method followed by previous study [24]. The transesterification was carried out refluxing glycerol and a N_2_ gas flow in a four-neck glass flask. Neem oil and glycerol were put in the glass batch reactor. The hydroxyl group molar ratio of neem oil to glycerol was 1: 2 upon stirring. Next, 0.2 wt% of CaO (by the weight of oil) was added to the reactants as a catalyst. Reaction time was kept for 3 h at 220 °C. The transesterification reaction is shown in Figure 2A [24]. 

### 2.2. Basic Properties of Plant Oils and Isocyanate

Castor oil and Desmodur N were used as received. The acid value and hydroxyl value of castor oil, neem oil and NOG, and the NCO content of isocyanate were estimated in the authors’ laboratory according to previously studied methods [25]. Molecular weight and weight distribution of neem oil and NOG were measured by a Hitachi L-6200A gel permeation chromatographer (Tokyo, Japan). The measurement followed the previous investigation [25]. The average hydroxyl content was calculated as follow [26]:

Average hydroxyl content (mmol/g) = Hydroxyl value (mg KOH/g) ÷ 56.1 (g/mol)(1)

### 2.3. Preparation of Bio-Based PU Foams

The PU foams were synthesized from castor oil, neem oil, NOG and Desmodur N. The raw material ratio is shown in Table 1. The hydroxyl molar ratios of castor oil and NOG were set to 10/0, 9/1 and 8/2. The neem oil was set to 8/2, the same weight as the reference group. NCO/OH molar ratios of isocyanate (Desmodur N) and the polyol (Castor oil and NOG) were set to 1.0, 1.5, and 2.0. The NCO/OH molar ratios of O1-2, O2-2 and O3-2 set as 1.3, 1.9 and 2.5 because neem oil was used to the same weight as NOG for comparison. The preparation steps of PU foam were as follows: Polyol and isocyanate were mixed in a cup with surfactant (DC5388), blowing agent (distilled water) and catalyst (DBTDL) and then stirred rapidly at 200 rpm for 1 min at room temperature. Then, the mixture was moved to a mold for casting. The cream time, the end of the rise time, the tack-free time and the height of the mixture were transcribed and determine the rate of reaction. The properties were measured after 1 week at room temperature. 

### 2.4. Properties of Neem Oil-Based PU Foams

#### 2.4.1. Density

The density of PU foams were measured according to ASTM D 1622. The density was calculated by the following equation:

Density (kg/m^3^) = Specimens’ weight (mg) ÷ Specimens’ volume (cm^3^) (2)

Five specimens were determined in this test. 

#### 2.4.2. FTIR

Fourier-transform infrared analysis (FT-IR, PerkinElmer spectrum 100) was used to confirm the structure of PU foams by the attenuated total reflectance (ATR) method. The analysis was performed within a spectral range of 650–4000 cm^−1^ at a resolution of 4 cm^−1^. 

#### 2.4.3. Optical Micrograph

USB digital microscope (UPMOST/UPG629) was used to observe the images of PU foams. Pore sizes were statistically analyzed by ImageJ. Open cell content was measured from the microscope images. According to previous investigation, the cell window status can be recognized as open, partially open and closed pores. The open content was calculated by the following equation [27]:

Open content (%) = (No + 0.5Np) ÷ (No + Nc + Np) × 100 (3)

where, No, Np and Nc are the numbers of open, partially opened and closed cells. 150 windows of a foam were determined in this test.

#### 2.4.4. Mechanical Properties

Following the ASTM C365-00 standard, specimens were cut into 2.54 × 2.54 × 1.27 cm^3^. The mechanical properties of vertical and parallel to the foaming direction were measured using a universal testing machine (Shimadzu EZ -500NSX) with a 5 mm/min load speed. The compressive strength corresponds to a stress at 10% and 25% strain. Specific compressive strength was calculated according to the following formula:

Specific compressive strength (kPa·m^3^/kg) = Stress (kPa) ÷ Density (kg/m^3^) (4)

#### 2.4.5. Water and Chemicals Resistance

The method followed by the previous study [23,25]. The foam specimens were cut into 1 × 1 × 1 cm^3^ for immersion testing. The solution used distilled water at 25 °C and 50 °C, and acetone, ethyl acetate and toluene at 25 °C for 1 h. Five specimens were determined in this test. 

#### 2.4.6. TG Analysis

TG analysis (TGA, PerkinElmer Pyris 1) set a heating rate of 10 °C/min from 50 °C to 600 °C in a N_2_ was carried out to measure the thermal stability of PU foams. 

#### 2.4.7. Statistical Analyses

We used SPSS (Software version 20, SPSS Inc., Chicago, IL, USA) for statistical analysis. The statistical significance of results was calculated by Scheffe’s test. Statistical results mark as English alphabet in the table and the bar graph. The *p*-value was 0.05.

## 3. Results and Discussion

### 3.1. Basic Properties of Plant Oils and Isocyanate

The acid value and hydroxyl value of castor oil, neem oil and NOG are shown in Table 2. The hydroxyl values of castor oil and neem oil are 150.9 mg KOH/g and 7.2 mg KOH/g, respectively. This is because the fatty acid of castor oil has a hydroxyl structure (Ricinoleic acid), while neem oil does not. Castor oil can react with isocyanate and form the urethane bond [12,23]. After the transesterification reaction of neem oil, the hydroxyl value of NOG was 281.5 mg KOH/g, which shows that NOG can also be used as a polyol. Figure 2B shows the variations in molecular weight distribution. The molecular weight distribution curves of neem oil and NOG were divided into two and one peaks. The primary fatty acids of neem oil were oleic acid (50%), stearic acid (30%) and palmitic acid (12%) [16]. The low molecular weight range may be due to the unsaturated oleic acid reacting with oxygen and then generating oxidation products [28]. Weight average molecular weight (M_w_), number average molecular weight (M_n_) and polydispersity of neem oil were 1125 g/mol, 602 g/mol and 1.9, respectively. After transesterification, M_w_, M_n_, and NOG’s polydispersity were 1134 g/mol, 707 g/mol and 1.6, respectively. Low polydispersity of NOG indicated that transesterification can help to narrow the molecular weight distribution of the polyol [29]. From the percentage of molecular weight, the NOG contents of monoglyceride and diglyceride were 68.7% and 21.6% respectively [30]. The average hydroxyl contents of castor oil, neem oil and NOG were 2.7 mmol/g, 0.1 mmol/g and 5.1 mmol/g. The results indicated that the average hydroxyl content of NOG was much higher than that of neem oil. 

### 3.2. The Basic Properties and Pore Structure of PU Foams

The foaming properties of PU foams are shown in Figure 2C–E. With increasing NCO/OH ratio, we found a difference in the tack-free time. The tack-free time decreased significantly because of high reactivity between the OH and NCO groups. This result is the same as previous study that showed that increasing NCO/OH ratio can shorten the foaming reaction time [32]. The results also showed that the end of rise time and tack-free time of neem oil-based PU foam were significantly longer than those of castor oil- and NOG-based PU foams. Without a hydroxyl structure, neem oil dilutes the reactants. The reaction time of bio-based PU foam can be adjusted between 5 min to 10 min, which is acceptable for laboratory and industrial manufacturing processes. 

**Figure 3:**
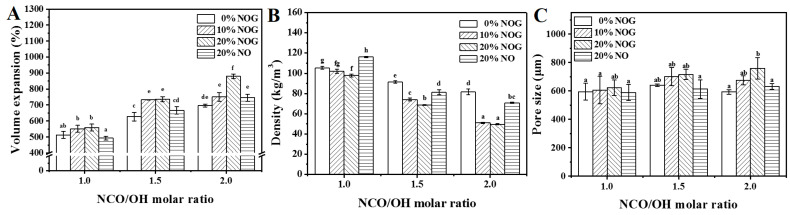
Volume expansion (**A**), density (**B**) and pore size (**C**) of PU foams. The NCO/OH molar ratios of O1-2, O2-2 and O3-2 set as 1.3, 1.9 and 2.5, respectively. The data analyzed using one-way ANOVA with Scheffe’s test, the different lowercase letters indicate statistical differences in the bar graph.

**Figure 4:**
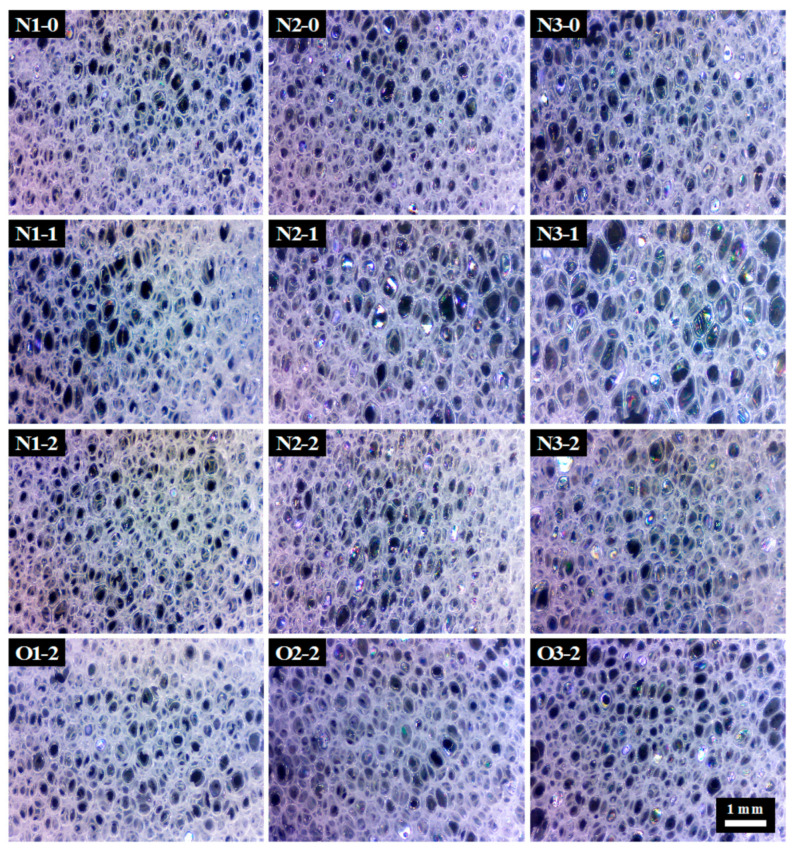
Pore micrographs of PU foams. All scale bars of subfigures are the same as O3-2.

As shown in Figure 3A,B, the volume expansion and densities of PU foams grow up and drop off with an increasing NCO/OH ratio, similar to the findings of previous studies [32]. The respective densities of N1-0, N1-1, N1-2, N2-0, N2-1, N2-2, N3-0, N3-1 and, N3-2 are 105.5, 102, 97.9, 91.5, 74.1, 68.7, 81.7, 51.0 and 49.7 kg/m^3^. The results, similar to previous studies, show that the increasing NCO/OH ratio decreases density [32]. The result also suggests that isocyanate reacts with water to release CO_2_ for the foaming stage [33] and the reaction generated heat helps expand the cell structure and leads to an increase in the volume expansion of the PU foam. These results also indicate that the density of neem oil-based PU foam is significantly higher than that of NOG-based PU foam. The densities of O1-2, O2-2 and, O3-2 are 116.2, 81.4 and 70.8 kg/m^3^, respectively. This result is due to NOG polyol with secondary hydroxyl groups which helps the PU resin to cure rapidly and maintain the foam’s structural integrity during the foaming process [34]. Figure 4 displays the PU foam micrographs. The pictures show an open-pore structure and elongated cellular structures in the foam rise direction. The open cell contents of N1-0, N1-1, N1-2, N2-0, N2-1, N2-2, N3-0, N3-1, N3-2, O1-2, O2-2 and O3-2 are 85.9%, 87.9%, 84.4%, 84.5%, 82.5%, 83.5%, 81.5%, 77.7%, 83.6%, 83.8%, 82.0% and 81.2%. Figure 3C shows that the range of pore sizes of PU foams is from 588.3 µm to 758.0 µm. The pore size of N3-2 is larger than those of the others significantly. The results indicated that a high NCO/OH ratio increases the pore size of PU foams because isocyanate reacts with water and generated CO_2_ bubble [33]. On the other hand, even though NCO/OH ratio of O1-2, O2-2 and O3-2 is higher than that of N1-2, N2-2 and N3-2, densities and pore sizes of neem oil-based PU foams are more heightened and smaller than those of NOG-based PU foams. These results suggest that neem oil is without a hydroxyl structure and dilutes the reactants to reduce gas production. The morphology also can prove the PU foams are homogenous and successfully prepared.

### 3.3. FTIR Spectra for PU Foams

**Figure 5:**
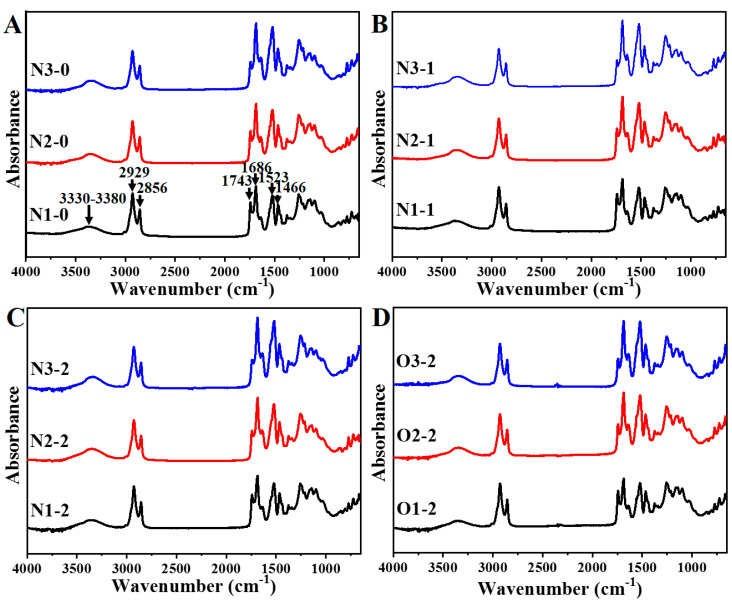
FT−IR spectra of 0% NOG (**A**), 10% NOG (**B**), 20% NOG (**C**) and 20% neem oil (**D**).

Figure 5 exhibits the FT-IR spectra of PU foams. NOG- and neem oil-based PU foams have a similar pattern as castor oil-based PU resin [12,23]. The region at 3330–3380 cm^−1^ assigned to the O–H and N–H stretching vibration. The peaks at 2929 cm^−1^ and 2856 cm^−1^ are attributed the methyl and methylene groups, respectively. The C=O stretching vibrations of free urethane, bonded urethane and bonded urea are ascribed to the peaks at 1743, 1686, and 1640 cm^−1^, respectively. The N–H bending vibration and the C–N stretching vibration for amide II assigned to at 1523 cm^−1^. The absorption intensity at 1258 cm^−1^ can be attributed to amide III. Table 3 shows the relative absorption intensities of bonded urethane, bonded urea, amide II, and amide III to free urethane. The results indicated that the NCO/OH ratio and NOG ratio increased with increasing ratios of amide II, amide III and bonded urea to free urethane. This can be attributed to a high NCO/OH ratio resulting in an increase of hard segment content, which led to increased absorption intensity [35]. Furthermore, secondary hydroxyl groups of NOG polyol reacted with isocyanate which increased stiffness of chain reduces chain mobility and increasing hard segment content [34]. Although NCO/OH ratios of O1-2, O2-2 and O3-2 are higher than that of N1-2, N2-2 and N3-2, the result indicated that adding neem oil reduces the relative absorption intensities of urea, amide II, amide III and urethane because of the dilution of PU resin. The FT-IR spectra data demonstrated that increasing the NCO/OH and NOG ratios could affect the structure of PU resins. 

### 3.4. Mechanical Properties of PU Foams

**Figure 6:**
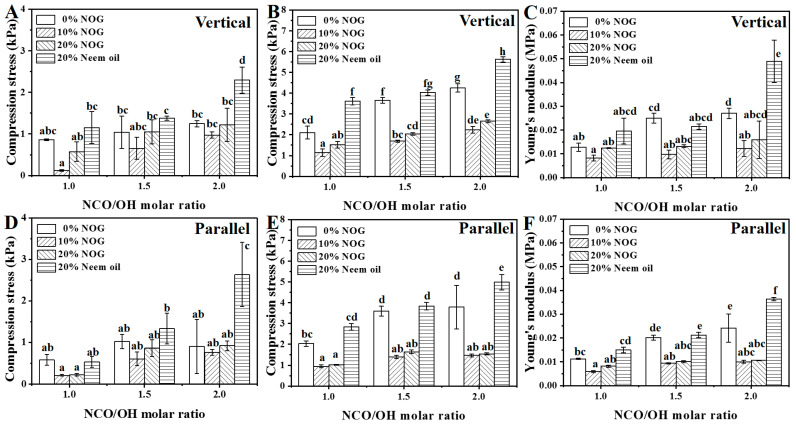
Compressive strength at 10% (**A**,**D**) and 25% (**B**,**E**) strain and young’s modulus (**C**,**F**) in the vertical and parallel directions. The NCO/OH molar ratios of O1-2, O2-2 and O3-2 set as 1.3, 1.9 and 2.5, respectively. The data analyzed using one-way ANOVA with Scheffe’s test, the different lowercase letters indicate statistical differences in the bar graph.

**Figure 7:**
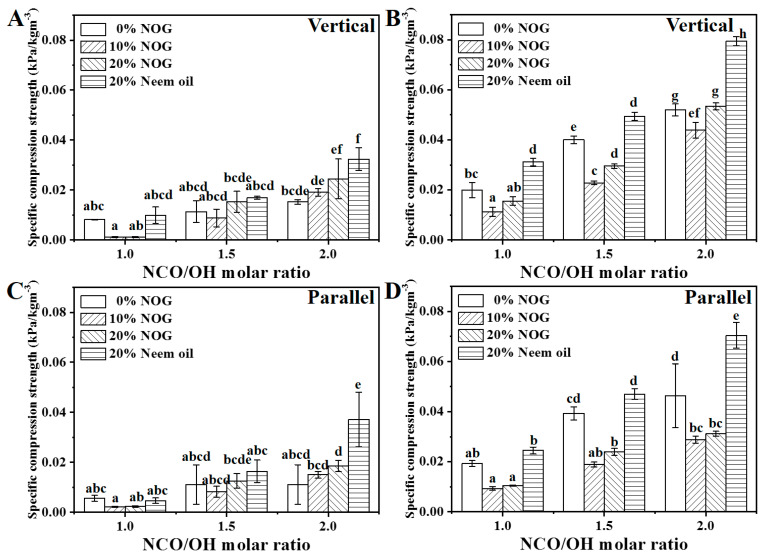
Specific compressive strength at 10% (**A**,**C**) and 25% (**B**,**D**) strain in the vertical and parallel directions. The NCO/OH molar ratios of O1-2, O2-2 and O3-2 set as 1.3, 1.9 and 2.5, respectively. The data analyzed using one-way ANOVA with Scheffe’s test, the different lowercase letters indicate statistical differences in the bar graph.

The upward growth direction of the PU foam during the foaming process is defined as the vertical direction. Figure 6 shows the mechanical properties in the vertical and parallel directions. The results indicated that compressive strengths at 10% and 25% strain and young’s modulus of vertical direction were slightly higher than those of the parallel direction. The result indicates that PU foams have anisotropy because the pores of PU foam elongated slightly in the foaming direction (Figure 4), which enhances the mechanical properties of PU foam in the vertical direction [36]. The specific compressive strength was determined as the compressive strength divided by PU foam density to normalize the mechanical properties [37,38]. Figure 7 shows the specific compressive strengths of the PU foams. The ranges of specific compressive strengths of PU foams were from 0.0082 to 0.0795 kPa·m^3^/kg and 0.0056 to 0.0704 kPa·m^3^/kg in the vertical and parallel directions, respectively. In previous studies, the specific compressive strength of PU foams reinforced with organic filler ranged from 0.0049 to 0.0073 kPa·m^3^/kg [39,40]. The specific compressive strengths of the bio-based PU foams were much higher than those of PU foams in previous studies [37]. When the NCO/OH ratios were set at 1.0 and 1.5, the results indicated that the specific compressive strength at 10% strain had no significant difference. In contrast, increasing the NOG ratio can increase the specific compressive strength at 25% strain. When that NCO/OH ratio was set as 2.0, increasing the NCO/OH ratios and neem oil can significantly increase the specific compressive strength at 25% strain. The results showed that specific compressive strength increases as polyol reacts with a twice-molar ratio of the isocyanate. This is because a high NCO/OH ratio increases the crosslinking and hard segment domain [41]. On the other hand, NOG can help react with isocyanate to increase the stiffness of the chain and improve the specific compressive strength in a low NCO/OH ratio [34]. The specific compressive strength at 25% strain of O2-2 is higher than that of N3-2 at NCO/OH ratio of ca. 2.0. The result indicates that neem oil can also help to enhance the specific compressive strength. The addition of 5% linseed oil can also improve slightly compressive strength. The results suggest that the fatty acid chain can be a natural modifier and help protect the structure against compressive stress [20]. The results demonstrate that NOG and neem oil can enhance the mechanical properties of PU foams.

### 3.5. Water and Solvents Resistance of PU Foams 

**Figure 8:**
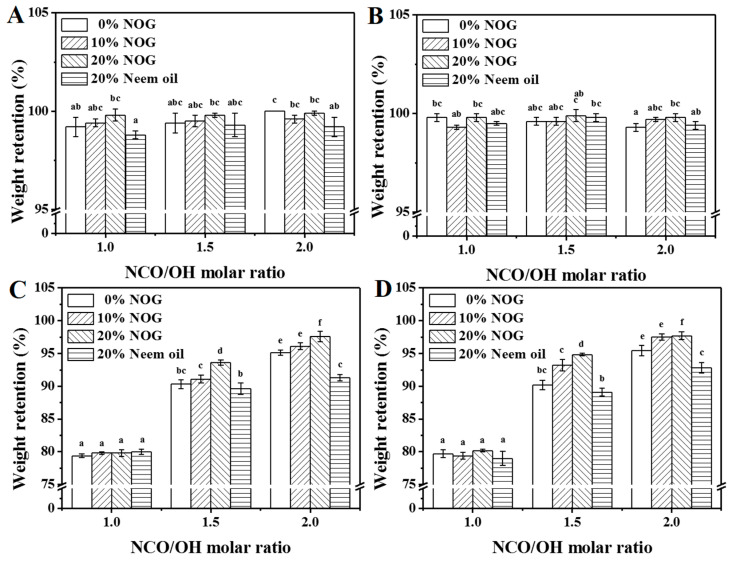
The weight retention of PU foams immersed in 25 °C (**A**) and 50 °C (**B**) distilled water, acetone (**C**) and ethyl acetate (**D**) for 1 h. The NCO/OH molar ratios of O1-2, O2-2 and O3-2 set as 1.3, 1.9 and 2.5, respectively. The data analyzed using one-way ANOVA with Scheffe’s test, the different lowercase letters indicate statistical differ-ences in the bar graph.

The weight retentions for PU foams in water, acetone and ethyl acetate are listed in Figure 8. The weight retentions are ca. 100% in water (Figure 8A,B). The results indicate that the bio-based PU foams are waterproof and can be used as commodities. In acetone and ethyl acetate, the weight retentions of PU foams are 79.4–97.6% and 79.0–97.7%, respectively (Figure 8C,D). The results indicate that PU foams with a higher molar ratio of NCO/OH were more resistant to organic solvent because of the high degree of crosslinking. NCO/OH ratios of O1-2, O2-2 and O3-2 are higher than that of N1-2, N2-2 and N3-2, solvents resistance of neem oil-based PU foams are lower than those of NOG-based PU foams. Notably, increasing the NOG ratio enhanced the solvent resistance significantly. The results suggested that the crosslinking density of the NOG-based PU foam was higher than castor oil-based and neem oil-based PU foams when that NCO/OH ratio set higher than 1.5. The solvents resistance of neem oil-based PU foams was significantly lower than those of NOG-based PU foams. This is because neem oil has no reactive hydroxyl group, which reduces the structure of urethane, urea, amide II and, amide III (Table 3). The previous study also indicated polyol with secondary hydroxyl groups can increase the crosslinking density of PU resin [34]. The results demonstrate that NOG can enhance the solvent resistance of bio-based PU foam.

### 3.6. Thermal Properties of PU Foams

**Figure 9:**
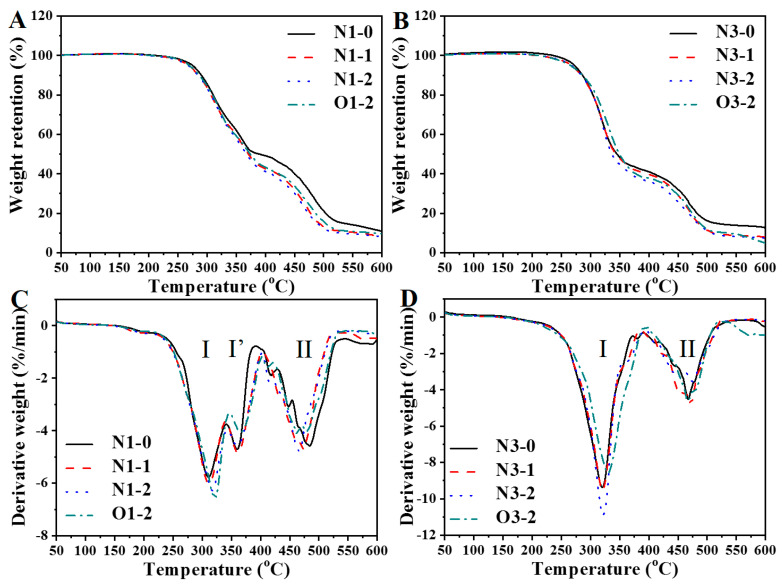
TGA (**A**,**B**) and DTG (**C**,**D**) curves for PU foams.

Figure 9 exhibits the TG curves and differential TG (DTG) curves of PU foams. According to the DTG curves, PU foams’ thermal decomposition can be divided into two or three thermal decomposition stages. Multiple decomposition steps occurred in PU polymers, similar to previous studies [12,23]. The thermal decomposition temperature occurred between 250 to 400 °C and presented multiple thermal patterns. The decomposition can be attributed to the degradation of the hard segment, including urea, urethane, biuret and allophanate structures [42]. The results show that increasing the NCO/OH molar ratio changed the thermal degradation from three stages to two stages and the onset temperature increased. Increasing the NCO/OH ratio led to formation of more urea structure and increased the intermolecular force between PU polymer chains (Table 3). At I stage, Table 4 shows the weight loss at high NCO/OH ratio is higher than that at low NCO/OH ratio. Increasing NCO/OH ratio also caused a side reaction and formed allophanate and biuret structures, which reduces the thermal stability and increased thermal weight loss [43]. A previous study indicated the TGA results for castor oil [44]. The result indicated that the temperature range of mass loss is around 300 to 420 °C. The thermal decomposition between 400 to 550 °C can be attributed to the degradation of the soft segments of castor and neem oils [23]. The results show that high NCO/OH ratio at synthesis of the foams causes low weight loss at stage II because the proportion of soft segments in PU decreases. The temperatures of neem oil- and NOG-based PU foams at 10% (T_10_) weight loss ranged from 283 to 290 °C. These results the PU foams had similar thermal stability. T_10_ of castor oil glyceride-based PU resin and TiO_2_ were ca. 250 °C in the previous study [45]. The results indicate that mixing NOG and neem oil with castor oil-based PU foams have good thermal stability because of the secondary hydroxyl groups and the oil-based soft segments [23,34].

## 4. Conclusions

In this study, castor oil-based PU foams were modified by neem oil and NOG. Castor oil and NOG were used as bio-polyol and Desmodur N with NCO/OH molar ratios of 1.0, 1.5 and 2.0. NOG is a narrow-distribution polyol. The average hydroxyl content of NOG was higher than that of castor oil and neem oil. The short and controllable reaction time of PU foam can be accepted in manufacturing processes. The results show that the end of rising time and tack-free time of NOG-based PU foams can be reduced significantly. NOG-modified PU foams show low density and excellent organic solvent resistance. In contrast, adding neem oil increases the density and mechanical properties but reduces the chemical resistance of PU foam. The mechanical properties of PU foams were reinforced with addition of NOG or neem oil at NCO/OH ratios of 1.0 to 1.5. Adding NOG and NCO/OH ratio affect the chemical structure and increase the hard segment content, resulting in enhanced mechanical properties. Increasing the NCO/OH molar ratio led to high weight retention in the chemical solvent resistance, indicating a better cross-linked structure. The TG analysis results show that NOG-based PU foams’ thermal stabilities are slightly lower than those of neem oil-based foams. All PU foams have excellent thermal stability. This result demonstrates that bio-based PU foams can be prepared with various properties by adjusting the molar ratios of NCO/OH, NOG and neem oil. Neem oil and its glyceride can act a nature modifier and bio-based polyol. The PU resins can be used as cushion foam [46] and a wood composite adhesive [12]. This investigation demonstrated the feasibility of the application of non-edible oils in the bio-based PU industrial process in the near future.

## Figures and Tables

**Table 1:** The weight of the raw materials set as to prepare bio-based PU foam.

Code	NCO/OH	Raw Materials (Parts by Weight)						
		Polyol			Isocyanate	Surfactant ^2^	Catalyst ^2^	Blowing Agent ^2^
		Castor Oil	NOG ^1^	Neem Oil				
N1-0	1.0	100	0	-	49	4.0	4.0	4.0
N1-1		100	6	-	54	4.3	4.3	4.3
N1-2		100	13	-	61	4.5	4.5	4.5
N2-0	1.5	100	0	-	73	4.0	4.0	4.0
N2-1		100	6	-	81	4.3	4.3	4.3
N2-2		100	13	-	92	4.5	4.5	4.5
N3-0	2.0	100	0	-	98	4.0	4.0	4.0
N3-1		100	6	-	109	4.3	4.3	4.3
N3-2		100	13	-	122	4.5	4.5	4.5
O1-2	1.3	100	-	13	61	4.5	4.5	4.5
O2-2	1.9	100	-	13	92	4.5	4.5	4.5
O3-2	2.5	100	-	13	122	4.5	4.5	4.5

^1^ NOG: Neem oil glyceride. ^2^ The addition was based on the total weight of the castor oil, NOG and neem oil.

**Table 2:** The basic properties of castor oil, neem oil and NOG.

Code	Acid Value (mg KOH/g)	Hydroxyl Value (mg KOH/g)	M_w_ ^2^ (g/mol)	M_n_ ^3^ (g/mol)	Polydispersity (M_w_/M_n_)	Average Hydroxyl Content (mmol/g)
Castor oil	2.1 ± 0.1	150.9 ± 4.6	1181 ^4^	1150 ^4^	1.0	2.7
Neem oil	7.2 ± 0	7.2 ± 0	1125	602	1.9	0.1
NOG ^1^	2.3 ± 0.2	283.8 ± 5.0	1134	707	1.6	5.1

^1^ NOG: Neem oil glyceride. ^2^ M_w_: Weight average molecular weight. ^3^ M_n_: Number average molecular weight. ^4^ The data measured by a previous study [31].

**Table 3:** Relative intensity of bonded urethane, bonded urea, amide II and amide III to free urethane.

Code	Relative Absorption Intensity *			
	Bonded Urethane (1686 cm^−1^)	Bonded Urea (1640 cm^−1^)	Amide II (1523 cm^−1^)	Amide III (1258 cm^−1^)
N1-0	1.75	0.30	1.32	1.37
N1-1	1.74	0.84	1.57	1.43
N1-2	2.32	1.15	2.19	1.75
N2-0	1.55	0.35	1.23	1.23
N2-1	2.03	0.87	1.83	1.53
N2-2	2.42	1.20	2.29	1.85
N3-0	1.54	0.66	1.32	1.32
N3-1	2.07	1.01	1.86	1.55
N3-2	2.40	1.21	2.28	1.80
O1-2	1.28	0.66	1.17	1.19
O2-2	1.84	0.98	1.76	1.47
O3-2	1.81	1.10	1.81	1.48

* Based on the absorption intensity of the free urethane bond at 1743 cm^−1^.

**Table 4:** Thermal parameters of PU foams.

Code	I				I’				II				T_10_^3^ (°C)	Char Yield ^4^ (%)
	Onset	Peak	WL ^1^	PH ^2^	Onset	Peak	WL	PH	Onset	Peak	WL	PH		
	(°C)	(°C)	(%)	(%/min)	(°C)	(°C)	(%)	(%/min)	(°C)	(°C)	(%)	(%/min)		
N1-0	275	310	36.6	5.8	355	360	17.1	4.7	445	484	26.2	4.6	290	10.9
N1-1	267	312	38.7	6.0	350	361	20.0	4.8	432	474	27.7	4.7	285	8.2
N1-2	272	318	43.2	6.3	351	360	22.2	4.6	433	466	24.8	4.8	284	8.1
O1-2	273	321	41.1	6.6	358	366	18.1	4.1	436	463	26.5	4.2	286	9.6
N3-0	278	320	61.2	9.4	-	-	-	-	432	468	21.9	4.5	286	12.7
N3-1	281	321	60.3	9.6	-	-	-	-	430	473	21.9	4.8	286	8.0
N3-2	285	322	60.6	10.9	-	-	-	-	423	474	27.1	3.6	283	7.4
O3-2	293	330	57.3	8.5	-	-	-	-	430	467	25.0	4.5	286	4.9

^1^ WL: Weight loss. ^2^ PH: Peak height. ^3^ T_10_: Temperature at 10% weight loss occurs. ^4^ Char yield: Char yield at 600 °C.

## Data Availability

The data presented in this study are available on request from the corresponding author.
